# Development of a composite healthy ageing score: evidence from middle-to-older aged Australians

**DOI:** 10.1093/heapro/daad043

**Published:** 2023-07-22

**Authors:** Eme Eseme John, Thomas Astell-Burt, Ping Yu, Chris Brennan-Horley, Xiaoqi Feng

**Affiliations:** Population Wellbeing and Environment Research Lab (PowerLab), Sydney, NSW, Australia; School of Health and Society, Faculty of Arts, Social Sciences and Humanities, University of Wollongong, Wollongong, Australia; Population Wellbeing and Environment Research Lab (PowerLab), Sydney, NSW, Australia; School of Health and Society, Faculty of Arts, Social Sciences and Humanities, University of Wollongong, Wollongong, Australia; Centre for IT Enabled Transformation, School of Computing and Information Technology, Faculty of Engineering and Information Sciences, University of Wollongong, Wollongong, Australia; School of Geography and Sustainable Communities, and Australian Centre for Culture, Environment, Society and Space (ACCESS), University of Wollongong, Wollongong, Australia; Population Wellbeing and Environment Research Lab (PowerLab), Sydney, NSW, Australia; School of Public Health and Community Medicine, Faculty of Medicine, University of New South Wales, Sydney, NSW 2052, Australia

**Keywords:** healthy ageing, healthy ageing score (HAS), functional capacity, resilience

## Abstract

We developed and validated a composite healthy ageing score (HAS) to address the absence of a definitive composite score comprising multiple health domains that measure healthy ageing in epidemiology. The HAS is developed from 13 health domains reported to influence healthy ageing. Data to measure these domains was extracted from the 45 and Up Study baseline. We applied best practices for scale validation and development. Physical functioning, cognitive function, mental health, sleep, quality of life, balance, social connections and overall health were retained. Functional capacity and resilience were uncovered as underlying latent structures. The HAS ranges from 0 to 16 with higher scores indicating a better health profile. This research contributes a comprehensive measuring tool, HAS, It enables examination and comparison of individual or collective health profiles and the investigation of the factors that influence their chances of living healthy for longer.

## Background

Ageing is an inevitable population change requiring worldwide public attention (Hermanova, 1995). Ageing can be thought of as a complex process of increased vulnerability to disease, adaptation to physical, social and psychological changes that span the life course (Tosato *et al.*, 2007). Ageing signs can differ between individuals. Therefore, focusing mainly on negative individual physiological outcomes neglects the vast experience of heterogeneity and outcomes that are coincident with ageing. Again, although disability may diminish some functional aspects, it is not always a barrier to fulfilment and active ageing.

Countries report mortality statistics to the World Health Organization (WHO) based on the International Statistical Classification of Diseases and Related Health Problems (ICD-10), which is useful for calculating life expectancy. (World Health Organization, 2015b). Although this was useful in calculating life expectancy for the different countries, the data did not report non-fatal debilitating health outcomes. Consequently, in 1980, the WHO issued the *International Classification of Impairments, Disabilities and Handicaps (ICIDH) as* a tool to classify the consequence of disease. However, researchers criticised the (ICIDH) for being too closely aligned with disease outcomes and its failure to acknowledge the influence of personal, social and environmental impacts on disability. Also, there was no distinction between features of a person’s health and the features of the experience of disability that are not aspects of health such as education or employment (Bickenbach *et al.*, 1999; Üstün *et al.*, 2003). In response, the WHO issued the ICIDH in 1980, but it was criticised for being too closely aligned with disease outcomes and for not acknowledging the influence of personal, social, and environmental impacts on disability. As a result, the World Health Assembly approved the International Classification of Functioning, Disability and Health (ICF) in May 2001 to address these limitations and provide a comprehensive classification system that includes personal and environmental factors (World Health Organization, 2001).

The ICF provides a valuable tool for researching disability in all its dimensions, including impairment of body functions or structures, societal-level activity limitations, and participation restrictions. It can also be used to understand and classify human functioning and disability at various levels in information systems for clinical research, policy development, and other public health uses. However, there are multiple reasons why a healthy ageing score (HAS) is needed when the ICF is the benchmark for classifying function and disability across the life course. First, the 1450 categories of the ICF make it prohibitive for daily practice. Researchers developed ICF Core Sets to describe the functioning, disability and health of specified subpopulations (Cieza *et al.*, 2004; Yen *et al.*, 2014). To date there is no healthy ageing ICF Core Set, and only one study has developed and validated the Geriatric ICF Core Set specifically for community-living older adults aged 75 years and older without dementia (Spoorenberg *et al.*, 2015; Karlsson and Gustafsson, 2022). Two other studies have proposed ICF Core Sets for geriatric patients in early post-acute rehabilitation facilities (Grill *et al.*, 2005) and community-dwelling elderly adults in primary care (Book *et al.*, 2020), but these require further testing and validation. Second, large sample studies on healthy ageing have not utilised the ICF in its present form. Instead, most studies separately investigated well-being in old age using morbidity, cardiometabolic factors, perceived well-being, mental health, cognitive function and physical capability (de Keijzer *et al.*, 2020). Some of these studies also treated environmental factors as influencers or predictors of healthy ageing rather than as a component of functioning and disability in old age. Hence exploring the existing data on healthy ageing and coalescing the various health outcomes in old age into a composite score is helpful. Therefore this study seeks to utilise the available data to ascertain the core components relevant to healthy ageing in Australia in the context of surrounding green space. (Australian Institute of Health and Welfare, 2003; Üstün *et al.*, 2003).

Introduced by the World Health Organization’s (WHO) Regional Office for Europe in 1990, ‘Healthy ageing’ is defined as ‘the process of developing and maintaining the functional ability that enables well-being in older age’, while functional ability is defined as ‘having capabilities that enable all people to be and do what they have reason to value (Hermanova, 1995; WHO, 2015). This concept comprises four aspects of healthy ageing: functional abilities (health-related attributes that allow people to do what they have reason to value); intrinsic capacities (all the physical and mental capabilities that an individual can draw on); environments (sociodemographic traits, the natural and built environment that form the context of a person’s life); and well-being (happiness, security and fulfilment). Positive relationships between those factors can benefit various aspects of health across our life span, and enhance our quality of in old age (Zadworna, 2022).

Healthy ageing depends on many interrelated factors, suggesting its optimal measure is likely to be a composite measure instead of any single domain. Presently, there is no consensus on a particular healthy ageing measure as different indices are used by different researchers across different disciplines and world regions (Lara *et al.*, 2013). Analogous reasoning has been applied by environmental epidemiologists as the ‘exposome’ concept, which describes the totality of environmental exposures that may enhance or diminish human health outcomes (Wild, 2012). Healthy ageing can be assessed and operationalised by different indicators, including morbidity, activities of daily living, mental health, cognitive function, physical capability, perceived well-being, cardio-metabolic risk, health behaviours and self-rated health (Vrijheid, 2014; Lu *et al.*, 2019; Nieuwenhuijsen *et al.*, 2019). Despite this, environmental epidemiologists have tended not to focus on analysing composite healthy ageing representations. This may be potentially due to a lack of definitive and validated healthy ageing metric.

A potential factor limiting the use of composite HASs is the absence of scores with validated psychometric properties. Sanders *et al*. developed a healthy ageing index which includes systolic blood pressure, pulmonary vital capacity, creatinine, fasting glucose, and Modified Mini-Mental Status Examination score for the American population (Sanders *et al.*, 2013). Between June 2011 and March 2012, the Chinese government conducted the baseline survey for the China Health and Retirement Longitudinal Study. Based on this study, Wu *et al*. constructed the Chinese Healthy Ageing Index, having six components: systolic blood pressure, peak expiratory flow, telephone interview for cognitive status, estimated glomerular filtration rate, fasting glucose, C-reactive protein (Wu *et al.*, 2018). In Europe, Jaspers *et al*. used data from the Rotterdam study starting in 1990 to develop a HAS and compared its difference across age and sex. The score included seven domains: chronic diseases, mental health, cognitive function, physical function, pain, social support, and quality of life. A total score (range 0–14) was constructed and was assessed continuously and in tertiles (13–14: healthy ageing, 11–12: intermediate ageing, 0–10: poor ageing) (Jaspers *et al.*, 2017). However, none of these studies provided validated psychometric properties of the composite scores they used.

However, there is a more accepted use of HASs in the related field of social epidemiology. For example, Lu *et al*. reviewed the measurement of healthy ageing used in 50 studies across 23 countries. They classified them into eight domains: physical capabilities, cognitive functions, metabolic and physiological health, psychological well-being, social well-being, general health status, health behaviours, and short-form health survey and health indices (Lu *et al.*, 2018). Despite the uneven deployment of the healthy ageing concept in different strands of epidemiology, it is important to treat healthy ageing as a measurable outcome for empirical validation and comparison. Nonetheless, there is neither an agreed standard to measure healthy ageing nor a definitive contextual discourse to establish the conceptual parameters. For example, the healthy ageing quiz developed by Australia’s National Ageing Research Institute did not report validated psychometric properties of the score. Further, measuring physical activity instead of physical functioning for the elderly misrepresents their capacity to do the activities they value and need to live independently. Another limitation of the HAQ was poor national representativeness and examination of its psychometric properties using a restricted sample of 297 participants (Cyarto *et al.*, 2013).

### Aim

The objective of this investigation is to develop a HAS by enhancing the applicability of the healthy ageing quiz domains in the context of environmental epidemiology in Australia. The improvement process involves the inclusion of additional domains and the elimination of overlapping or redundant ones, guided by sound scale development and validity principles. Furthermore, the study endeavours to identify the most relevant domains that contribute to the construction of the HAS. In addition, any underlying structures in the HAS will be examined through confirmatory factor analysis. The proposed HAS is expected to add to the knowledge of healthy ageing.

### Ethics statement

The conduct of the 45 and Up Study was approved by the University of New South Wales Human Research Ethics Committee (HREC). The University of Wollongong & ISLHD Health and Medical Human Research Ethics Committee and the UOW Social Sciences Human Research Ethics Committee approved this project (Ethics number 2016/158, 2020/206).

## METHOD

### Approach

The proposed HAS incorporates the relevant domains of health that impact the capacity of individuals to lead lives that they find fulfilling as they age. The HAS construction started with 13 domain items that are sequentially eliminated based on their intra, inter and item-total correlations. It coalesced these 13-item contributions into as few items as possible, which capture the most variation. The reliability of the score was assessed using Cronbach-alpha. Confirmatory factor analysis was used to identify underlying latent constructs in the scale. Socioeconomic, demographic and geographic variations in the HAS were also investigated.

### Data

Data for this study were from The Sax Institute’s 45 and Up Study based on the population of New South Wales (NSW), Australia. 267 153 participants joined the study by completing a baseline questionnaire (between January 2006 and December 2009), gave signed consent for follow-up, and linked their information to routine health databases. The 45 and Up is a large-scale collaborative Australian cohort study of individuals over 45 years, which provides researchers with timely and reliable information about public health of the middle-aged and the elderly in Australia. The study was over-sampled by a factor of two, individuals over 80 years and living in rural areas. Recruitment into the 45 and Up Study started in February 2006, and in 2007, 100 000 participants joined the study, with a total 250 000 joining by the end of 2009.

### Domain identification, item generation, content validity and expert evaluation

The 13 domains that form this study have been measured using already validated scales used in peer-reviewed studies and international standards. The initial domains are chronic diseases/comorbidity, physical functioning, smoking, BMI, diet and nutrition, alcohol intake, cognitive function, mental health, sleep, quality of life, balance and falls, social connections and overall health. Participants were assigned a score of 2 if they exceeded the recommended benchmark for a given domain, 1 if they met the benchmark, and 0 if they did not meet the recommendation (Table 1). The HAS is the sum of the retained domain scores (A1), with higher scores indicating a better health profile.

**Table 1:** Healthy ageing score domains, construction and properties

S/N	Healthy ageing score domains	Domain scoring	*N*	Mean	SD
1.	Physical functioning	2: Score 70+ on the SF36 1: Score between 50 and 70 0: Score below 50	216 247	1.75	0.002
2.	Cognitive function	2: ‘excellent’ or ‘very good” memory 1: ‘good’ memory 0: ‘fair’ or ‘poor’ memory	257 918	1.27	0.001
3.	Mental health	2: K10 score of 10 to 19 1: K10 score of 20 to 29 0: K10 score of 30 +	197 577	1.87	0.001
4.	Sleep	2: 6 to 10 hours of sleep 0: Less than 6 hours or more than 10 hours of sleep	259137	1.89	0.001
5.	Quality of life	2: ‘excellent’ or ‘very good’ QOL 1: ‘good’ QOL 0: ‘fair’ or ‘poor’ QOL	252636	1.88	0.001
6.	Balance and falls	2: 0 falls in last 12 months 0: 1 or more falls in the last 12 months	252 952	1.64	0.002
7.	Social connection	2: Score 10 and above on DSSI 1: Score between 7 and 9 on DSSI 0: less than 7 on DSSI	142 101	1.79	0.001
8.	Overall health	2: ‘excellent’ or ‘very good” overall health 1: ‘good’ overall health 0: ‘fair’ or ‘poor’ health	257 465	1.38	0.001
**Total: revised healthy ageing score (HAS)**		**0–16**	**80 826**	**13.89**	**0.008**

### Missing cases

At the domain level, participants with incomplete information for measuring each domain item were dropped. While calculating the total score, participants who had one or more domain scores missing were also dropped. At the level of calculating the HAS, participants missing any domain item score were also dropped. A total of 207 192 (76%) entries were dropped while computing the base HAS, whereas 186 327 (70%) entries were dropped while computing the revised HAS, indicating that an additional 20 865 participants were used to compute the revised HAS.

### Item reduction analysis

An item reduction analysis was performed to ensure that the score was parsimonious, internally consistent and contained only domains that made unique contributions. From a numerical perspective, more models with fewer parameters may be easier to fit because the fit function is minimised over a lower-dimensional space in which the global minimum may be easier to locate (Raykov and Marcoulides, 1999).

Initially, item-test correlations were computed to determine the degree of association between individual items and the total score of the scale items. To address poor fitting, item-total correlations, which examine the association between each item and the summation of the remaining items, excluding itself, were also examined (Howard and Forehand, 1962). Items with moderate or strong correlations with the adjusted items-total correlations are `retained. Small item-total correlations are evidence the items are not measuring the same construct as the other items included. A correlation value less than 0.2–0.3 indicates that the corresponding item does not correlate very well with the scale overall and, thus, may be dropped (Everitt and Skrondal, 2002). Boateng *et al*. recommended adjusted items-total correlations <0.3 were less desirable and should be dropped (Boateng *et al.*, 2018). Domain items with desirable correlations and whose removal in the scale caused the alpha value to drop were retained. Statistical analyses were performed in Stata v16.

### Item reduction, factors and dimensions

A scale should have a high level of internal consistency which is measured by the Cronbach-alpha (Boateng *et al.*, 2018). Items that reduced the Cronbach-alpha when eliminated from the scale are considered for retention. Factor analysis was used to investigate the unobserved continuous dimensions along which participants differ. It also uses eigenvalues to establish the optimal number of latent factors reflected by the domain items retained in the scale. Latent factors with eigenvalues greater than 1 and valid values of uniqueness are considered, and domain items with factor loadings of over 0.4 load uniquely on the individual latent factors are retained (Boateng *et al.*, 2018). Factors with eigenvalues greater than 1 with valid AIC (Akaike information criterion) and BIC (Bayesian Information Criterion) will be retained. The scree plot of factor loadings will also be examined for bends to provide evidence of the dimensionality of the HAS construct and the point after which the plot flattens considerably points to its dimensionality.

### Reliability and confirmatory factor analysis

**Fig. 1:**
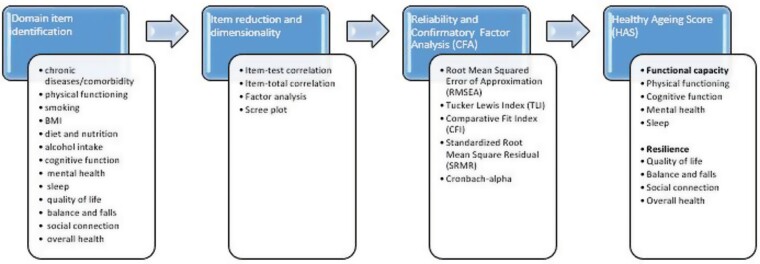
Development of the healthy ageing score (HAS).

Cronbach-alpha of the retained items was calculated as a measure of reliability. The retained items and factors were modelled using confirmatory factor analysis and the model diagnostics indicated how suitably the retained items are factored into the latent structures. The process of developing the HAS is summarised in Figure 1.

## RESULTS

### Extraction of items

As shown in Table A1 in the appendix, physical functioning, cognitive function, mental health, sleep, quality of life/optimism, balance and falls and overall health were retained having met the criteria of adjusted item-test, item-rest correlations and effect on the Cronbach-alpha. Removing social connections from the score reduced the Cronbach-alpha marginally (0.504) so it was retained. In addition, the scree plot and factor loadings plot were examined. The bend after the second point in the scree plot indicates that the retained domain items measuring the HAS construct were in two dimensions because after the second factor the plot flattens considerably (A2—appendix). Also, the AIC and BIC values were only valid for the two-factor model (factor 1 AIC, BIC: 6652, 6769; factor 2 AIC, BIC: 3660, 3885) as all others were invalid.

Based on these results, it is evident the HAS comprises two latent structures. The first structure measures spatial awareness and functional capacity required to perform activities of daily living and maintain overall health. The proposed domains measuring this factor are physical functioning, cognitive function, balance and falls and overall health. We also propose that the second latent structure is the complement of the first. It measures the latent factor manifesting as resilience through recreation/relaxing and social engagement, improving the quality of life. The proposed items measuring this factor are mental health, quality of life and social connections.

### Confirmatory factor analysis

Seven items were retained: physical function, cognitive function, balance and falls, overall health, mental health, QOL and social connections. In the factor analysis, two factors were retained since the third factor was reported as a boundary or Heywood case. To identify which domains are captured in the two latent constructs, we considered the effect of the items to be in terms of improving functional capacity or psychophysiological stress recovery. Therefore, we considered the domains physical functioning, cognitive function, balance and falls, and overall health as measuring the latent construct—physical functioning mental health, quality of life and social connections as measuring the resilience construct.

**Fig. 2:**
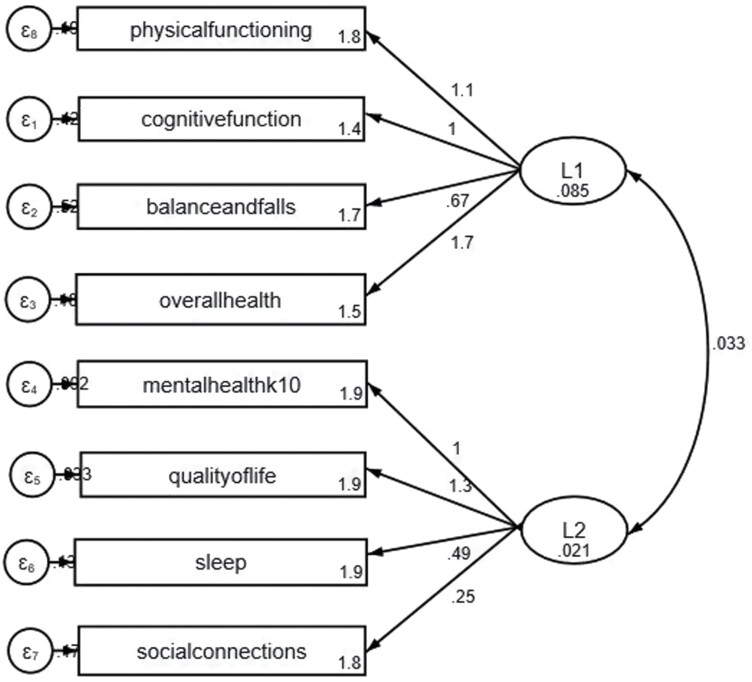
Structural equation model (SEM) of the healthy ageing score (HAS) and its latent structure.

To test if the two-dimensional latent structure scale is valid, we conducted a confirmatory factor analysis using the proposed latent variable structure (Latent variable 1: physical functioning, cognitive function, balance and falls and overall health; Latent variable 2: mental health, quality of life, sleep and social connections). A structural equation model (SEM) of the hypothesised model was fit (Figure 2). Examination of the model fit indices in Table 2 showed the proposed model fairly described the underlying latent structure since the RMSEA, TLI, CFI and SRMR are within the acceptable threshold. The model fit indices were within the acceptable threshold (RMSEA = 0.056, TLI = 0.919, CFI = 0.95, SRMR = 0.027) though the model could be improved based on the RMSEA = 0.056 (Boateng *et al.*, 2018). The Cronbach-alpha was 0.6522 and indicated a modest fit. To improve the model, sleep was added to the resilience latent construct as it met item-total correlation criteria though its factor loading (0.3) was less than 0.4 as recommended. After including sleep, the TLI, CFI and Cronbach-alpha were unchanged. The model improved marginally as RMSEA reduced by 0.007 to 0.049 and SRMR reduced by 0.001 to 0.026 indicating the model fitted more closely to the true underlying latent structure in the data.

**Table 2:** Structural equation model (SEM) diagnostics

Model fit indices	Threshold^**^	Model 1	Model 2
Root mean squared error of approximation (RMSEA)	RMSEA ≤ 0.05 0.05 ≤ RMSEA ≤ 0.08 RMSEA ≥ 0.10	0.056	0.049
Tucker lewis index (TLI)	TLI ≥ 0.90 TLI ≥ 0.95	0.919	0.919
Comparative fit index (CFI)	CFI ≥ 0.95	0.95	0.95
Standardised root mean square residual (SRMR)	SRMR ≤ 0.08	0.027	0.026
Cronbach-alpha	0.70 ≤ alpha ≥ 0.95	0.65	0.65

^**^(Boateng, Neilands *et al.* 2018).

The CFA output examination shows the retained items were significantly factored into their respective latent structures. For the functional capacity construct, the retained items were a good fit; physical functioning (β = 1.798, CI: 1.794, 1.802), cognitive function (β = 1.358, CI: 1.354, 1.363), balance and falls (β = 1.662, CI: 1.656, 1.667), overall health (β = 1.507, CI: 1.503, 1.512), *p* = 0.000 in all cases. The retained items for the resilience construct were also a good fit; mental health (β = 1.909, CI: 1.906, 1.911), quality of life (β = 1.938, CI: 1.936, 1.940), sleep (β = 1.931, CI: 1.928, 1.933), social connections (β = 1.790, CI: 1.787, 1.793). From the results, the root mean squared error of approximation (RMSEA) (0.049), Tucker Lewis index (TLI) (0.964) and comparative fit index (CFI) (0.964) are all within the acceptable threshold (Table 2) (Boateng *et al.*, 2018).

### Test of reliability

Cronbach’s alpha was calculated to evaluate the internal consistency of the items retained in the scale. The Cronbach-alpha of both models is approximately 0.7 and is, therefore, an adequate fit. For a limited number of items, a moderate Cronbach alpha between 0.6 and 0.7 is acceptable (Taber, 2018). Including sleep in the model improved the RMSEA from 0.056 to 0.049. Hence sleep was retained in the model. The final retained domain items are shown in A1 of the appendix.

## Discussion

The results show that a HAS can be developed based on eight items and can be further partitioned into two latent structures: a functional capacity dimension (using measures of physical functioning, cognitive function, balance and falls and overall health) and a resilient dimension (using measures of mental health, quality of life, sleep and social connections). This study is the first assessment of the healthy ageing construct in Australia to empirically validate an extended-item HAS and its reliability on a large representative sample of the Australian population. As noted earlier, a cause for concern in the healthy ageing quiz was the appropriateness of specific instruments for measuring particular health outcomes in older people. A case in point is physical activity. While it is crucial to assess the physical capacity of the elderly, it is inappropriate to do so in terms of vigorous activity measurements as engagement in vigorous physical activity tends to diminish with age (Milanović *et al.*, 2013). A more relevant and relatable measure would be measuring their capacity to function and carry out activities of daily living. From the literature, additional domains that contribute and are relevant to the health and well-being outcomes of the elderly, like physical functioning, BMI, mental health, quality of life and overall health, were included in this study. The aim was to coalesce the domains into as few items as possible while measuring the bulk if not all the variation in the healthy ageing construct. Three health domains that were reported to influence healthy ageing were added to the 10 domains of the healthy ageing quiz and the measures of these domains for participants of the 45 and Up study were extracted. The additional domains are physical functioning, mental health and overall health.

Mental health, quality of life, physical functioning, cognitive function and overall health were the additional domains included in the HAS and were measured using validated instruments. After the score validation analysis, the following five of the eight domains of the HAQ were dropped from the final HAS: physical activity, smoking and alcohol use, weight (BMI), diet and chronic conditions. Of the eight retained items, only three (balance and falls, sleep and social connections) are from the HAQ.

Physical activity tends towards sports and recreation while physical functioning represents older people’s capacity to execute activities of daily living. Therefore, the latter was preferred. Studies have shown that physical activity predicts high physical functioning in community-dwelling older adults. Men and women who were physically active at recommended levels were more likely to report high physical functioning at follow-up than their more sedentary counterparts (Hillsdon *et al.*, 2005; Halaweh *et al.*, 2017). Smoking and alcohol use were dropped from the HAS, indicating these domains are less relevant to the aggregate well-being of the participants. This could be a result of the reducing rates of smoking and alcohol use in Australia (Health and Welfare, 2020a, 2020b). Two in three Australian adults are overweight or obese, and 38% of adults over 45 years are obese (Health and Welfare, 2019). It is plausible that as long as BMI does not affect physical functioning, it was not regarded as necessary in the HAS. Chronic diseases did not make it into the recommended HAS confirming the earlier position that for the participants, resilience and adaptation to physical, social and psychological changes, even with the presence of disease, are more reliable indicators of health and well-being. Interestingly physical activity, smoking and alcohol consumption were omitted in the HAS, yet these are three key behaviours that medical practionioners tell us to modify to keep healthy. These results in no way imply that we ignore the advice, we submit that the HAS, and the resilience and physical functioning subdomains, are downstream outcomes of these health-related behaviours.

The results suggest that the present HAS is a distinct measure of healthy ageing which evolved from the analysis of as many relevant health domains as possible to a subset of health domains that the statistical procedures indicate are critical for healthy ageing. It followed a rigorous scale development procedure to refine the scale to its optimal dimensions while identifying the relevant latent structures. As this process was not done for the HAQ, we posit that the present HAS is an improvement on the HAQ and the full-item HAS. For instance, no Cronbach-alpha was reported for the HAQ, alpha for the full-item HAS is 0.51 and alpha for the recommended HAS is 0.65. The full item version will be retained to corroborate any findings made using the recommended scaled-down version as it had improved model diagnostics and the additional domain items are redundant. Measures of the items comprising our HAS can be adapted from available health surveys and used to compute the HAS for comparison across regions.

In addition to the contributions of socioeconomic circumstances in early childhood and institutional bias, the factors influencing health outcomes in later life are multifaceted. They include sociocultural, historical, demographic, genetic and environmental influences, as well as early childhood circumstances and institutional biases (Seabrook and Avison, 2012; Sadana *et al.*, 2016). The local environment plays a crucial role in promoting healthy ageing and HAS measurements can help highlight the extent of this contextual dependency for policymakers and researchers. The age-friendly environments in Europe (AFEE) project was established to work with cities and communities towards policy formulation and implementation of AFEE, which can increase opportunities for health and well-being for older people in their local environments. The AFEE project identified eight domains of age-friendly action that require adaptation of the physical environment, social environment, and municipal services to promote healthy ageing in place. These domains include outdoor environments, transport and mobility, housing, social participation, social inclusion and non-discrimination, civic engagement and employment, communication and information, and community and health services (World Health Organization, 2015a).

Improving outdoor environments can enhance neighbourhood walkability, access to public spaces and buildings, and feelings of safety. Interventions in the transport and mobility domain aim to promote safe, accessible, appropriate, and reliable transport services and infrastructure for active living, enabling people to maintain mobility, independence, and connections as they age. Adequate, accessible, safe, and affordable housing is a goal of interventions in the housing domain, along with support for ageing in place through measures that modify existing housing and build new ones better adjusted to older people’s needs. Social participation, social inclusion and non-discrimination, civic engagement and employment, communication and information, and community and health services are other domains that require interventions to create environments that are socially inclusive and provide opportunities for engagement in political, economic, and public life. These interventions include promoting engagement in paid employment, volunteering activity, local decision-making, and providing timely, reliable, and understandable information about the community and available services.

### Limitations and implications

Due to the unavailability of matching data, the HAS is yet to be compared for convergence with any score developed to measure the same healthy ageing construct. The present study was conducted using data from Australians and, as such, its generalizability might need to be further examined in other countries or regions. There could also be a temporal variation in the responses of participants in the baseline survey as well as the waves of the survey. Healthy ageing cannot be measures independent of the local environment and facilities. Also, responses collected in the summer or during bush fire seasons may differ from those collected in winter, spring or autumn. Similarly, since the data used for developing this score was collected before the COVID-19 pandemic, participants could prioritise the relationship and well-being impact of the domain items differently during and after the pandemic. The influence of the neighbourhood age-friendliness on the HAS should be explored.

Future research may investigate how the HAS varies over time and across its domain items to generate insight into the level of influence of the healthy ageing components. It is also necessary to develop and validate a Healthy Ageing Core Set framed using the ICF that is applicable across all regions of the world. The influence of the COVID-19 pandemic on the HAS and its domain items also needs to be understood, as are the wider social, cultural, political and environmental determinants that generate, perpetuate and aggravate inequities in healthy ageing that the HAS can help play a role in identifying.

## Conclusion

This research proposed, developed and validated a HAS measurement tool, which contributes a data-driven method for measuring the health profile of the study population. Eight domains were shown to be crucial to healthy ageing and they contribute to two latent constructs: functional capacity and resilience. The proposed healthy ageing score adds to the body of knowledge and researchers might also use this data, or the method as a general guide for developing their own HAS versions in different countries or regions. A two-pronged approach that includes improving the functional capacity of the elderly to live independently and improve resilience appears to influence their well-being the most.

The two-dimensional healthy ageing structure, functional capacity and resilience, is important for policymakers to consider in planning public health interventions that support healthy ageing. In conclusion, promoting healthy ageing requires a multifaceted approach that takes into account the complex interplay of factors influencing health outcomes in later life. HAS measurements and the AFEE project’s eight domains of age-friendly action can help policymakers and researchers to understand the contextual dependency of healthy ageing and create age-friendly environments that maximise opportunities for health and well-being for older people in their local environments (World Health Organization, 2016, 2017, 2018).

## Supplementary Material

daad043_suppl_Supplementary_Appendix
