# Supplementary material for: Development of a composite healthy ageing score: evidence from middle-to-older aged Australians
Source: Health Promot Int. 2023 Jul 22;38(4):daad043. doi: 10.1093/heapro/daad043 (PMC10682690; doi:10.1093/heapro/daad043)
Supplement: daad043_suppl_Supplementary_Appendix [file daad043_suppl_supplementary_appendix.docx]

| **S/N** | **Healthy Ageing Score Domains** | **Domain Scoring** | **N** | **Mean** | **SD** |
| --- | --- | --- | --- | --- | --- |
| 1. | Physical Functioning | 2: Score 70+ on the SF36  1: Score between 50 and 70  0: Score below 50 | 216,247 | 1.75 | 0.002 |
| 2. | Cognitive Function | 2: "excellent" or 'very good" memory  1: "good" memory  0: "fair" or "poor" memory | 257,918 | 1.27 | 0.001 |
| 3. | Mental Health | 2: K10 score of 10 to 19  1: K10 score of 20 to 29  0: K10 score of 30 + | 197,577 | 1.87 | 0.001 |
| 4. | Sleep | 2: 6 to 10 hours of sleep  0: Less than 6 hours or more than 10 hours of sleep | 259,137 | 1.89 | 0.001 |
| 5. | Quality of Life | 2: "excellent" or "very good" QOL  1: "good" QOL  0: "fair" or "poor" QOL | 252,636 | 1.88 | 0.001 |
| 6. | Balance and Falls | 2: 0 falls in last 12 months  0: 1 or more falls in the last 12 months | 252,952 | 1.64 | 0.002 |
| 7. | Social Connection | 2: Score 10 and above on DSSI  1: Score between 7 and 9 on DSSI  0: less than 7 on DSSI | 142,101 | 1.79 | 0.001 |
| 8. | Overall Health | 2: "excellent" or 'very good" overall health  1: "good" overall health  0: "fair" or "poor" health | 257,465 | 1.38 | 0.001 |
| **Total: Revised Healthy Ageing Score (HAS)** | | **0 - 16** | **80,826** | **13.89** | **0.008** |

**A1. Revised Healthy Ageing Score Domains, Construction and Properties**


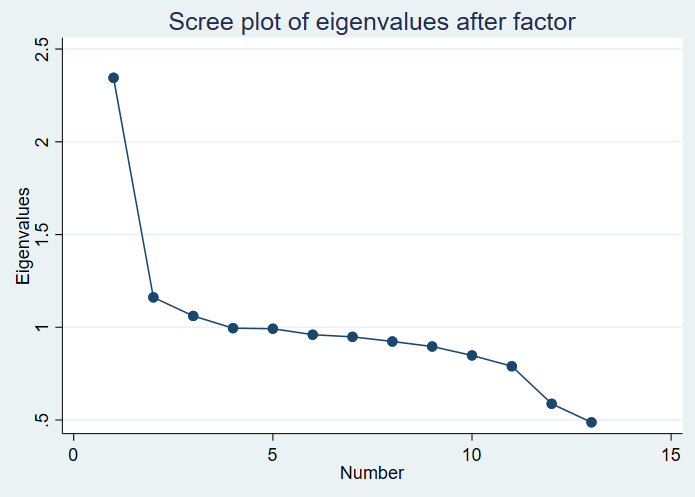


**A2. Scree plot of the HAS**
